# Cranberry Flavonoids Modulate Cariogenic Properties of Mixed-Species Biofilm through Exopolysaccharides-Matrix Disruption

**DOI:** 10.1371/journal.pone.0145844

**Published:** 2015-12-29

**Authors:** Dongyeop Kim, Geelsu Hwang, Yuan Liu, Yifei Wang, Ajay P. Singh, Nicholi Vorsa, Hyun Koo

**Affiliations:** 1 Biofilm Research Labs, Department of Orthodontics and Divisions of Pediatric Dentistry & Community Oral Health, University of Pennsylvania, Philadelphia, Pennsylvania, United States of America; 2 Department of Plant Biology and Plant Pathology, Rutgers University, New Brunswick, New Jersey, United States of America; 3 Philip E. Marucci Center for Blueberry and Cranberry Research and Extension, Rutgers University, Chatsworth, New Jersey, United States of America; Oregon Health & Science University, UNITED STATES

## Abstract

The exopolysaccharides (EPS) produced by *Streptococcus mutans-*derived glucosyltransferases (Gtfs) are essential virulence factors associated with the initiation of cariogenic biofilms. EPS forms the core of the biofilm matrix-scaffold, providing mechanical stability while facilitating the creation of localized acidic microenvironments. Cranberry flavonoids, such as A-type proanthocyanidins (PACs) and myricetin, have been shown to inhibit the activity of Gtfs and EPS-mediated bacterial adhesion without killing the organisms. Here, we investigated whether a combination of cranberry flavonoids disrupts EPS accumulation and *S*. *mutans* survival using a mixed-species biofilm model under cariogenic conditions. We also assessed the impact of cranberry flavonoids on mechanical stability and the *in situ* pH at the biofilm-apatite interface. Topical application of an optimized combination of PACs oligomers (100–300 μM) with myricetin (2 mM) twice daily was used to simulate treatment regimen experienced clinically. Treatments with cranberry flavonoids effectively reduced the insoluble EPS content (>80% reduction vs. vehicle-control; p<0.001), while hindering *S*. *mutans* outgrowth within mixed-species biofilms. As a result, the 3D architecture of cranberry-treated biofilms was severely compromised, showing a defective EPS-matrix and failure to develop microcolonies on the saliva-coated hydroxyapatite (sHA) surface. Furthermore, topical applications of cranberry flavonoids significantly weaken the mechanical stability of the biofilms; nearly 90% of the biofilm was removed from sHA surface after exposure to a shear stress of 0.449 N/m^2^ (vs. 36% removal in vehicle-treated biofilms). Importantly, *in situ* pH measurements in cranberry-treated biofilms showed significantly higher pH values (5.2 ± 0.1) at the biofilm-apatite interface vs. vehicle-treated biofilms (4.6 ± 0.1). Altogether, the data provide important insights on how cranberry flavonoids treatments modulate virulence properties by disrupting the biochemical and ecological changes associated with cariogenic biofilm development, which could lead to new alternative or adjunctive antibiofilm/anticaries chemotherapeutic formulations.

## Introduction

Biofilms are the prevailing microbial lifestyle in natural niches, causing many infectious diseases in humans [1]. Among them, dental caries is one of the most prevalent and costly biofilm-dependent oral diseases worldwide [2]. Cariogenic biofilms develop as pathogens accumulate on tooth surfaces, forming highly structured microbial communities that are tightly adherent and enmeshed in an extracellular matrix [3]. Exopolysaccharides (EPS), e.g. glucans, are key components in the cariogenic biofilm matrix, and are recognized virulence factors involved in the pathogenesis of dental caries [4–6].

Within the complex oral microbiome, *Streptococcus mutans* is not always the most abundant organism. However, this bacterium can rapidly orchestrate the formation of cariogenic biofilms when exposed to sucrose via EPS synthesis by *S*. *mutans*-derived glucosyltransferases (Gtfs) present on the tooth-pellicle and bacterial surfaces [4]. EPS formed *in situ* facilitate local accumulation of *S*. *mutans* (via membrane-associated glucan-binding proteins) while embedding them in a diffusion-limiting polymeric matrix [4]. In parallel, sugars are fermented by bacteria within the biofilm matrix, creating highly acidic microenvironments [7–10].

*S*. *mutans* can rapidly adapt to environmental stresses [11] that enhance its ability to thrive in these low-pH niches, ensuring virulent biofilm accretion and acid-dissolution of adjacent teeth [12]. Importantly, the EPS-matrix creates cohesive biofilms that are firmly attached to surfaces while protecting the embedded pathogens against antimicrobials, making them difficult to treat or remove [13,14]. Hence, biofilm-control approaches that disrupt EPS production, and thereby compromise the ability of *S*. *mutans* to assemble and maintain biofilms on tooth surfaces could be potentially effective alternatives to antimicrobials.

Cranberries are particularly rich sources of bioactive flavonoids such as flavonols and proanthocyanidins (PAC; flavan-3-ols) [15,16]. Cranberry extracts have been recognized for their anti-adhesion and anti-biofilm properties against several bacterial pathogens, including oral bacteria such as *Porphyromonas gingivalis* and *S*. *mutans* [17,18]. The major disruptive effects of cranberry flavonoids against cariogenic biofilms are on sucrose-dependent, EPS-mediated mechanisms [17,19]. We have demonstrated that cranberry PAC oligomers with specific degree-of-polymerization (DP4 and DP8-13) and flavonols (e.g. myricetin) are highly capable of inhibiting EPS production by Gtfs and impairing EPS-mediated *S*. *mutans* adhesion onto apatitic surfaces [19–21].

Previous and preliminary studies indicate that the combination of myricetin with PAC (particularly DP4 and DP9) may be more effective in reducing *S*. *mutans* Gtfs activity and exert enhanced anti-biofilm effects than each of the compounds alone without affecting bacterial viability [19–21]. However, these studies were conducted either in planktonic bacteria or using simple single-species biofilms. The present work investigates how an optimized combination of cranberry bioactives thwarts the ability of a bacterial oral pathogen (*S*. *mutans*) to assemble biofilms in the presence of other oral species using a mixed-species model that mimics the ecological and biochemical changes associated with cariogenic biofilm development [10]. Our data reveal the potential of cranberry flavonoids to control virulent properties of the biofilm by modulating EPS-matrix assembly and microbial composition that disrupt the mechanical stability and the pH at the biofilm/apatite interface.

## Materials and Methods

### Preparation of cranberry flavonoids

**Fig 1:**
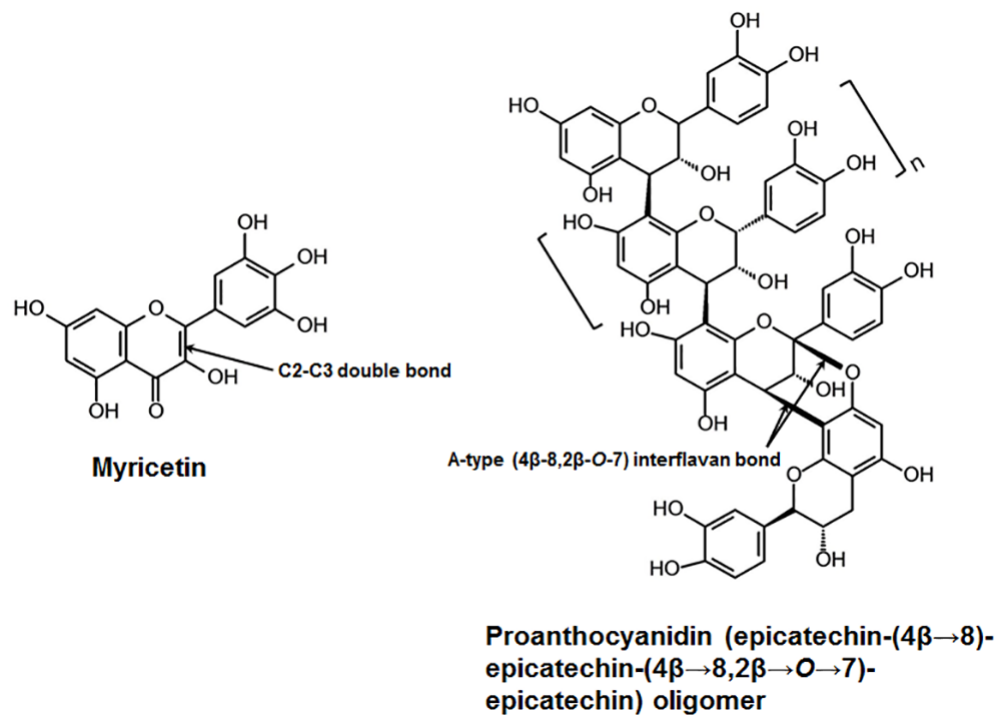
Structure of cranberry-derived flavonoids for combination therapy. Myricetin, one of the most active cranberry flavonols, is characterized by the presence of an unsaturated double bond between C2 and C3 and three hydroxyl-groups in the B ring. Proanthocyanidins (PACs) in cranberry are predominantly found in oligomeric forms (up to 13 monomeric units) with at least one A-type double interflavan linkage [epicatechin-(4β→8, 2β→*O*→7)-epicatechin] between the lower or lowest two units of the oligomer. The degree-of-polymerization (DP) is variable depending on the number of monomeric units; DP4 (tetramer) and DP9 (nonamer) are among the most abundant and bioactive cranberry PAC.

Cranberry flavonoids were derived from the fruit variety ‘Stevens’, which was harvested at PE Marucci Center, Rutgers University, Chatsworth, NJ. The variety ‘Stevens’ is a genetically well-defined and widely-grown commercial cranberry cultivar with high flavonoid content [22,23]. Individual flavonoid PACs were isolated and purified using previously published method with high purity (>95%) as determined using LC-MS-MS and MALDI-TOF-MS [20,21,24]. In this study, we focused on PACs tetramer (DP4) and nonamer (DP9), and flavonol myricetin based on their high bioactivity and abundance in cranberries. The combination and concentrations of the agents (300 μM DP4, 100 μM DP9 and 2 mM myricetin) were selected based on extensive optimization from our previous published and unpublished results [19–21]. The chemical structures of the cranberry bioactives are shown in Fig 1.

### Mixed-species ecological biofilm model and treatment regimen

**Fig 2:**
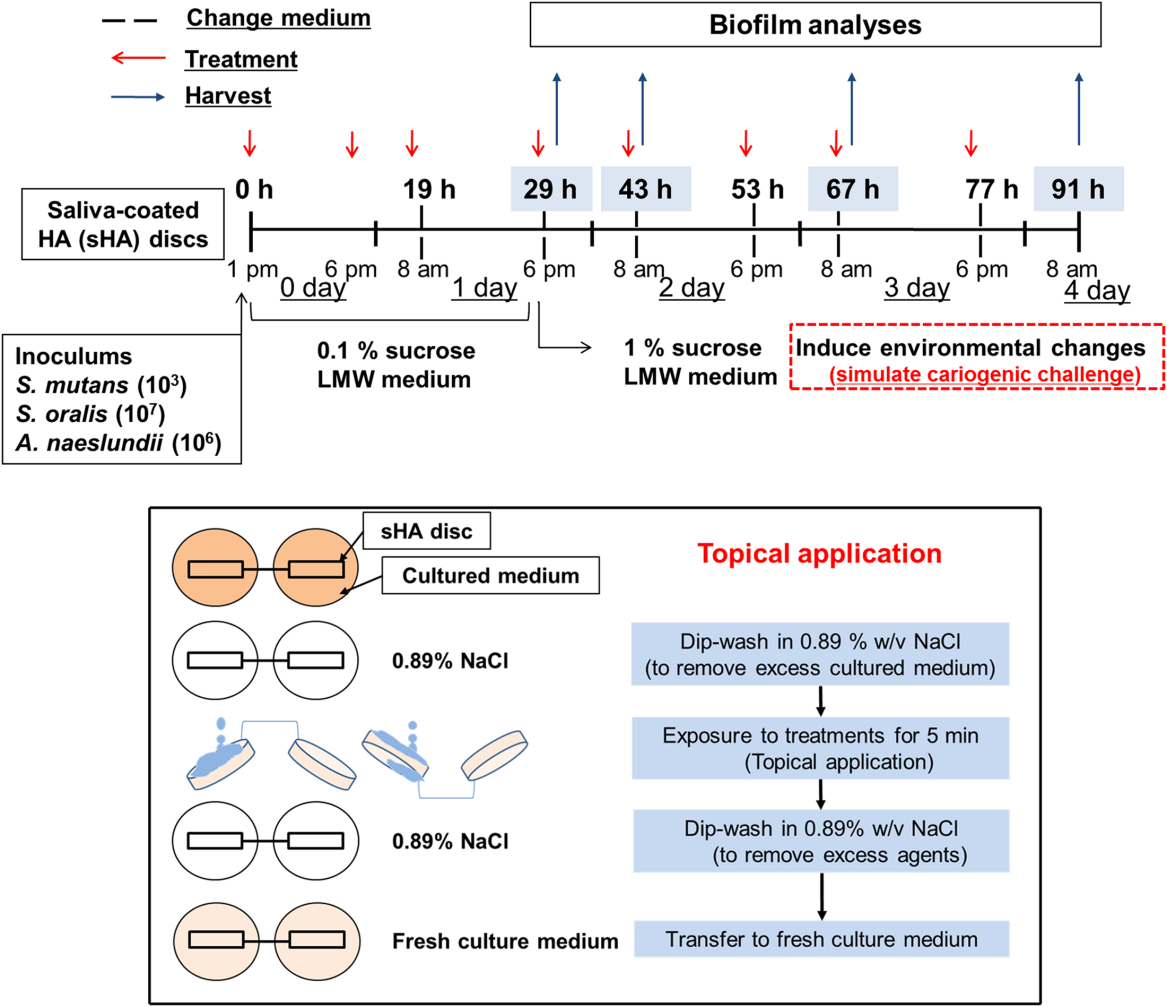
Experimental design for topical treatment regimen. Mixed-species cariogenic biofilm model and treatment regimen of selected combination of cranberry flavonoids (CranFlav). CranFlav or vehicle was topically applied twice daily with 5 min exposure.

The biofilm method used in this study was designed to mimic the formation of cariogenic biofilms according to the “ecological-plaque” concept [25] as detailed previously [10]. Briefly, hydroxyapatite discs (surface area of 2.7 ± 0.2 cm^2^; Clarkson Chromatography Inc., South Williamsport, PA, USA) were coated with filter-sterilized, clarified human whole-saliva for 1h at 37°C [19]. Early colonizers (*Actinomyces naeslundii* ATCC12104 and *Streptococcus oralis* ATCC 35037) and a well-established cariogenic streptococcus (*Streptococcus mutans* UA159; ATCC 100610) were grown in ultrafiltered yeast-tryptone extract broth (UFTYE; 2.5% tryptone and 1.5% yeast extract, pH 7.0) with 1% glucose at 37°C and 5% CO_2_ to mid-exponential phase [10]. Each of the bacterial suspensions were then mixed to provide an inoculum with a defined microbial population of *S*. *mutans* (10^3^ CFU/mL), *A*. *naeslundii* (10^6^ CFU/mL) and *S*. *oralis* (10^7^ CFU/mL), which are critical for the reproducibility of our model. The mixed population was inoculated in 2.8 mL of UFTYE containing 0.1% (w/v) sucrose, and incubated for 19 h to form an initial biofilm community on the sHA surface. Then, the biofilms were transferred to UFTYE containing 1% sucrose to induce environmental changes to simulate a cariogenic challenge. The culture medium was changed twice daily (8 am and 6 pm) until the end of the experimental period (91 h) (Fig 2).

The combination of PACs (DP4 and DP9) and myricetin was dissolved in the vehicle solution comprised of 5 mM phosphate buffer solution containing 20% EtOH and 0.8% DMSO (v/v; final pH 6.0). The combination of agents (referred to as CranFlav hereafter) was topically applied to the surface of sHA discs and the biofilms twice-daily (8 am and 6 pm) with 5 min exposure as described in Fig 2. First, 200 μL of the test agents (or vehicle) were applied topically to the surface of sHA discs using a pipette and kept for 5 min before placing the discs vertically in the culture medium. Then, a second treatment (with the test agents or vehicle) was performed after 5 h of biofilm development. Subsequently, the biofilms were treated twice daily (8 am and 6 pm) at 19, 29, 43, 53, 67 and 77 h of the experimental period. After each treatment, sHA discs were dip-washed in sterile saline solution (0.89% w/v NaCl) to remove excess agents, and then transferred to fresh culture medium (Fig 2). The retention of cranberry flavonoids following topical applications was estimated based on the Folin-Denis’s colorimetric method with some modifications [26] (S1 Fig).

### Three-dimensional biofilm architecture, quantitative analysis and *in situ* pH measurements

The effects of CranFlav treatments on the 3D architecture, EPS/bacteria accumulation and pH within intact biofilms were determined using our established protocols optimized for confocal biofilm imaging and quantification [10,27]. The EPS was labeled using 1 μM Alexa Fluor 647-labeled dextran conjugate (10 kDa; 647/668 nm; Molecular Probes Inc., Eugene, OR, USA), while the microbial cells were stained with 2.5 μM SYTO 9 (485/498 nm; Molecular Probes Inc.) [10]. Confocal imaging was performed using two-photon laser scanning microscope (SP5, Leica Microsystems, Buffalo Grove, IL, USA), and the image stacks acquired and processed as detailed by Xiao et al. [10]. The confocal images were analyzed via COMSTAT for the quantification of EPS and bacteria within intact biofilms, while Amira 5.4.1 software (Visage Imaging, San Diego, CA, USA) was used to create 3D renderings of the biofilm architecture [10,27]. Biochemical analysis of EPS and changes in the microbial population were also determined using standard colorimetric and viable cell counting methods [10,27]. Briefly, the biofilms were removed from sHA discs and homogenized by probe sonication (30-s pulse at 15% amplitude, equivalent to an output of 7 W); Branson Sonifier 150, Branson Ultrasonics, Danbury, CT, USA) in a sterile 0.89% (w/v) NaCl solution. The homogenized suspension was used to determine the amount of insoluble and soluble extracellular-polysaccharides by the phenol-sulfuric acid method using glucose as standard [27]. Further, the number of viable cells was determined by plating on blood agar using an automated spiral plater to determine the total number of viable cells (colony forming units (CFU)) per biofilm. The three species were differentiated by observation of colony morphology as well as microscopic examination [10]. To assess the biofilm pH, we used fluorescent pH-indicator Lysosensor yellow/blue (Molecular Probes Inc.) labeling method [10]. Briefly, the biofilms were incubated with Lysosensor yellow/blue-labeled dextran conjugate, and the pH values within intact biofilms were measured based on fluorescence intensity ratios of the dual-wavelength fluorophore. Lysosensor yellow/blue exhibits a dual-emission spectral peak (450 and 520 nm) that is pH dependent. The fluorescence intensity of both emission wavelengths and their ratio (I450/I520) within each biofilm image was measured using Image J 1.22. The ratios of fluorescence intensity of selected areas within each biofilm image were converted to pH values using the titration curves of ratios vs. pH (ranging from 3.5 to 7.0) as described previously [10].

### Biomechanical stability of biofilms

The mechanical stability of the biofilms treated with or without CranFlav was compared using a custom built device [14]. Biofilms were exposed to constant shear stress of 0.184 and 0.449 N/m^2^ for 10 min, which is capable of gradually removing *S*. *mutans* biofilm from sHA surfaces using our biofilm model [14]. The amount of remained biofilm dry-weight (biomass) before and after application of shear stress for each condition (vehicle- and CranFlav-treated) was determined.

### Statistical analysis

Values are expressed as mean ± standard deviation. A pairwise comparison was made between vehicle and CranFlav using a *t*-test by SPSS 18.0 software (IBM Co., Armonk, NY, USA). Differences are considered significant with p values <0.01.

## Results

### Topical treatments with cranberry flavonoids affect biofilm accumulation and 3D architecture

**Fig 3:**
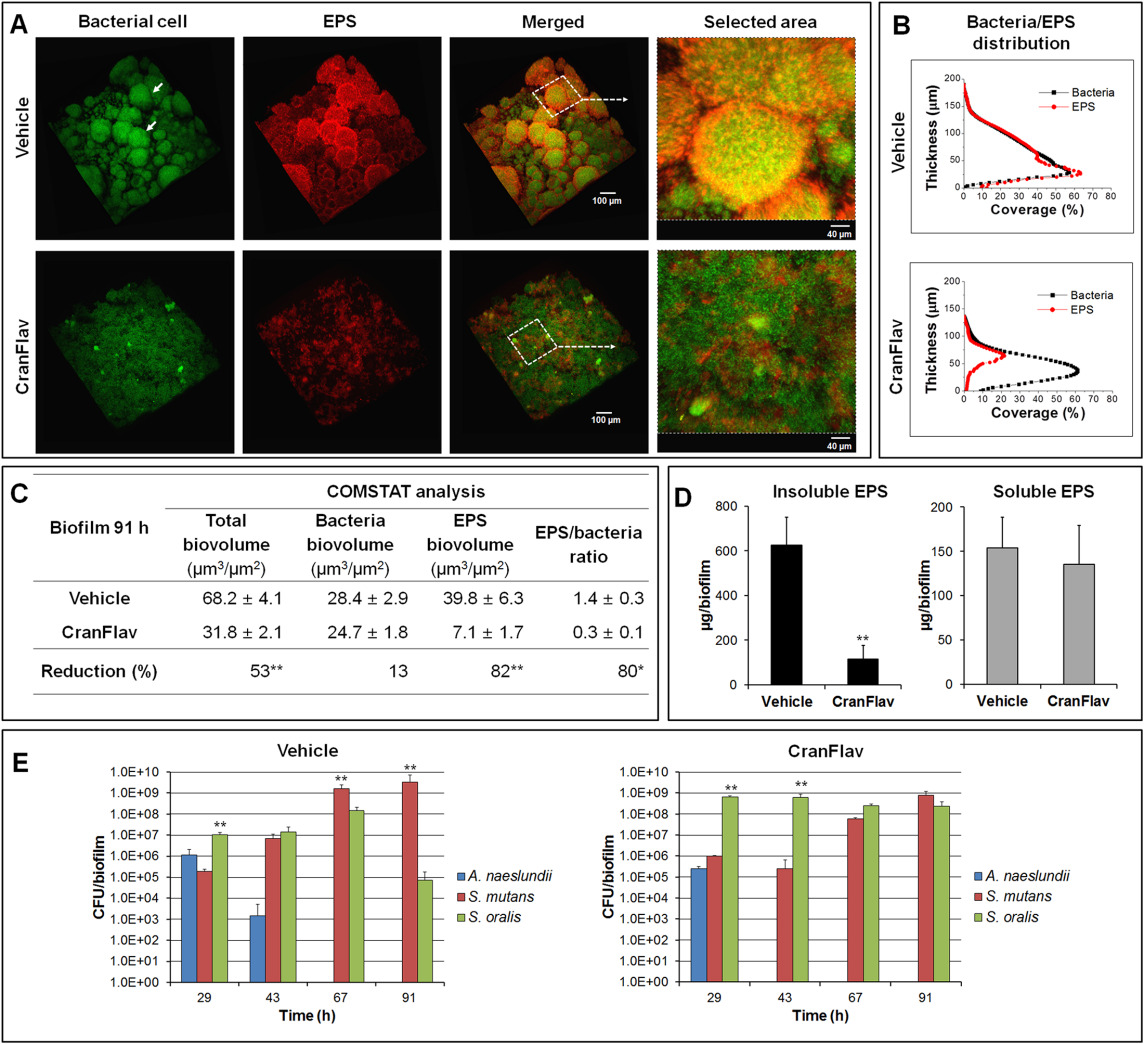
Three-dimensional (3D) architecture, EPS content and microbiological composition of mixed-species biofilms treated with CranFlav. (A) Representative 3D rendered images of mixed-species biofilms following topical treatments. Selected areas in panel (A) show detailed views of merged confocal images of bacterial cells (green) and EPS (red). (B) COMSTAT analysis of bacterial cells and EPS distribution across biofilm thickness (from the disc surface to the fluid phase interface). (C) Quantitative analysis of confocal images using COMSTAT software for determination of total, bacteria and EPS biomass (as well as EPS/bacteria ratio). The asterisks indicate that total biovolume, EPS biovolume and EPS/bacteria ratio of biofilm treated with CranFlav are significantly different from vehicle-control. A pairwise comparison between vehicle-control and CranFlav was conducted using a *t*-test. Values are significantly different from each other at *p*<*0.01 or **p<0.001. (D) Biochemical quantification of water-soluble EPS and insoluble EPS via colorimetric (phenol-sulfuric) assay (n = 6). A pairwise comparison between vehicle and CranFlav was conducted using a *t*-test. Values are significantly different from each other at **p<0.001. (E) Dynamics of microbial populations changes of mixed-species biofilms as determined by viable cell (colony forming units; CFU) counting (n = 6). A pairwise comparison between *S*. *oralis* and *S*. *mutans* was conducted. Values are significantly different from each other at **p<0.001.

We initially assessed the bioactivity of different combinations of the most active and abundant cranberry flavonoids (PAC tetramer and nonamer, and the flavonol myricetin) as determined previously [20,21]. Preliminary experiments have shown that the combination of these flavonoids (CranFlav) is more effective in disrupting EPS synthesis by GtfB and *S*. *mutans* accumulation within biofilms than either compound alone (S2 Fig). Thus, we evaluated the impact of CranFlav on EPS-mediated biofilm development and *S*. *mutans* survival using a cariogenic mixed-species biofilm model under cariogenic challenge [10]. CranFlav was applied topically twice-daily to simulate the treatment regimen that might be experienced clinically as illustrated in Fig 2. Using this model, we determined that CranFlav were retained within biofilms over time following topical treatments (S1 Fig), and caused major disruption in the structure (Fig 3A and 3B) and composition of the biofilms (Fig 3C–3E) without biocidal effects against neither planktonic nor biofilm-embedded bacteria (S3 Fig).

Confocal images of vehicle-treated biofilms show a well-developed EPS-matrix (in red) and the presence of bacterial cell clusters or microcolonies (in green; arrows in Fig 3A), as observed in sucrose-grown *S*. *mutans* biofilms [10,28]. The close-up image of the selected area reveals bacterial cells enmeshed and surrounded by EPS, forming densely packed and highly-structured microcolonies. Results from quantitative COMSTAT analysis show that most of the bacterial cells are co-localized with EPS (Fig 3B) across the biofilm thickness.

In sharp contrast, treatments with CranFlav resulted in a marked (albeit not completely) impairment of EPS-matrix development (Fig 3A and S4 Fig). COMSTAT analyses show >5 times less EPS in biofilms treated with CranFlav (vs. vehicle treated biofilms) but similar total bacterial biomass, which drastically reduced bacteria/EPS ratio (Fig 3C). Further biochemical assays show that the amount of water insoluble EPS was significantly reduced by CranFlav, but soluble EPS amount was unchanged (vs. vehicle-control; Fig 3D). Such effects can influence the pattern of bacterial binding and accumulation as insoluble EPS produced by the Gtfs promote cell-clustering and microcolony development [10]. Indeed, the fluorescence images show that bacterial cells were unable to form any microcolonies in CranFlav-treated biofilms. Higher magnification images of the selected area show sparse EPS amounts randomly interspersed among bacterial cells without any structural organization with the bacterial cells (Fig 3A), resulting in reduced bacteria-EPS co-localization (Fig 3B).

### Dynamics of *S*. *mutans* proportion changes within biofilms following CranFlav treatments

Concomitantly, we observed that treatments with CranFlav also influenced the proportion of the bacterial species during mixed-species biofilm development. In our biofilm model, the initial bacterial community on the sHA surface is comprised mainly of *S*. *oralis* and *A*. *naeslundii* (early colonizers), with *S*. *mutans* as the least abundant species. In the vehicle-treatment group, the proportion of *S*. *mutans* rapidly increased, becoming the dominant species within 67h-old biofilms, while *A*. *naeslundii* and *S*. *oralis* viable population decreased over time (Fig 3E). In contrast, CranFlav treatments greatly disrupted *S*. *mutans* colonization and further accumulation, resulting in a biofilm population dominated by *S*. *oralis* for most of the experimental period.

The microbiological data together with biofilm structure and EPS content characterization indicate that CranFlav effectively disrupt EPS-rich matrix assembly and the development of microcolonies while thwarting *S*. *mutans* outgrowth within mixed-species biofilms under cariogenic challenge (sucrose exposure). In turn, these biological actions could affect biofilm mechanical stability and the pH at the surface of attachment.

### CranFlav-mediated biological changes affect properties at the biofilm-apatite interface

#### Mechanical Stability

The biofilm cohesiveness and surface attachment strength are dependent on EPS-matrix and the formation of microcolonies [14,29], both of which are markedly impaired by CranFlav treatments. Thus, we investigated whether these structural changes could facilitate biofilm removal using a custom built device that produces shear forces to detach biofilms from the sHA surface [14].

**Fig 4:**
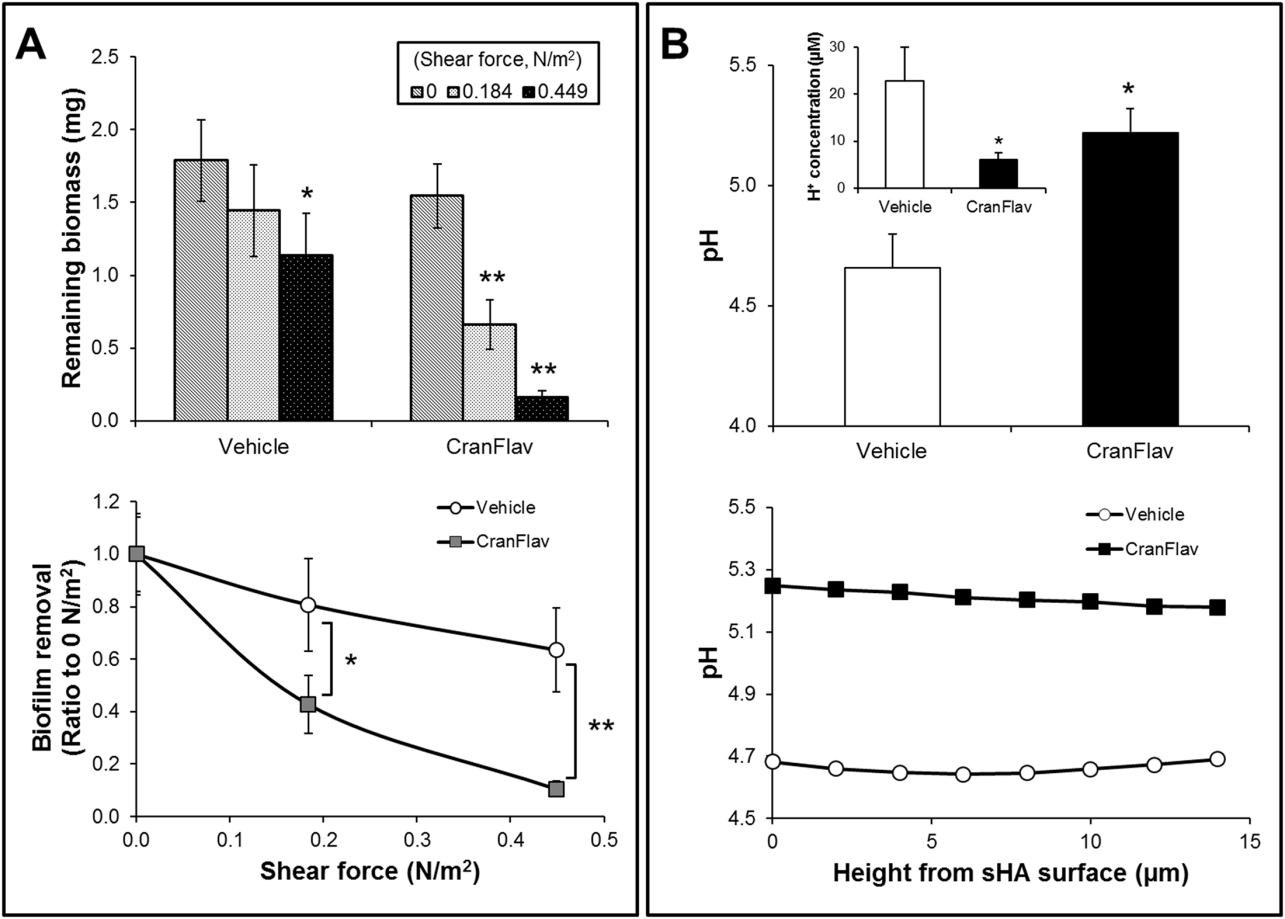
Mechanical stability and *in situ* pH within intact mixed-species biofilms treated with CranFlav. (A) The amount of remained biofilm dry-weight before and after application of shear stress was measured to determine the mechanical stability and attachment strength of CranFlav (or vehicle)-treated biofilms on sHA surface. We also determined biofilm removal at 0.184 and 0.449 N/m^2^ (vs. no shear, 0 N/m^2^) (n = 8). (B) *In situ* biofilm pH values were determined every 2 μm from the sHA surface, and averaged based on pH measurements across the biofilm interface (n = 3). The molar concentration of hydrogen ion (H^+^) at the biofilm/sHA interface (10–15 μm from the sHA surface) was estimated via the equation: [H^+^] = 10^-in situ pH^. Values are significantly different from each other at *p*<*0.01 or **p<0.001.

The ability of treated biofilms to withstand mechanical removal under shear stress was determined by measuring the amount of biofilm that remained on the sHA before and after shearing (Fig 4A). When exposed to high shear stress (0.449 N/m^2^), only a small portion of the biofilms treated with vehicle-control was removed (~35%), while at low shear force (0.184 N/m^2^) no significant effects were observed (Fig 4A). Strikingly, CranFlav-treated biofilms were nearly completely removed from sHA surface (~90% removal) at 0.449 N/m^2^; even low shear stress was capable of more than 50% biofilm removal. The data clearly demonstrate that CranFlav treatments weakened the overall biofilm structure and attachment strength, which facilitated its mechanical clearance from sHA surface when exposed to shear force.

#### *In situ* pH of Treated Biofilms

The EPS-enmeshed microcolonies act as diffusion-limiting barriers that help to create and maintain highly acidic pH (pH 4–4.5) at the surface of biofilm attachment [10], which is the hallmark for enamel demineralization and caries initiation [12,30]. Because CranFlav treatments prevent microcolony development, we have focused on measuring the pH values at the biofilm/sHA interface (10–15 μm from the sHA surface) as acidic pH at the surface of biofilm attachment is critical for caries development [31]. The results reveal that pH values in CranFlav-treated biofilms (5.22 ± 0.09) were significantly higher compared to those treated with vehicle-control (4.65 ± 0.14), indicating more than 5-fold less hydrogen ion concentration at the biofilm/sHA interface (p<0.01, Fig 4B).

## Discussion

Chemotherapeutic agents that interfere with the biofilm assembly process by virulent species and compromise its physical integrity under topical exposure regimen could effectively control pathogenic biofilms without indiscriminately killing commensal bacteria in the mouth [25]. Cranberries offer a rich source of bioactive flavonoids that could be useful for the development of alternative anti-biofilm and caries preventive therapies [17,19,32]. Advances in chemical separation-purification methods and the availability of well-characterized cranberry fruits have led to feasible and reproducible isolation of bioactive anti-biofilm flavonoids in high (mg) quantities [20,22], while removing inactive compounds (without anti-biofilm activity) such as anthocyanins [17]. Here, we have developed an optimized combination of cranberry flavonoids (CranFlav) that effectively hinders the biochemical and ecological changes associated with cariogenic biofilm development.

Cranberry flavonoids primarily target *S*. *mutans* Gtfs-mediated mechanism at the biofilm-pellicle interface [17,19]. Gtfs adsorbed to the pellicle produce glucans that promote local *S*. *mutans* colonization and accumulation while providing an insoluble matrix that facilitates cariogenic biofilm formation [4]. However, inhibition of surface-Gtfs is highly challenging as these enzymes undergo conformational changes when adsorbing to apatitic surfaces [33], rendering them resistant to common enzyme inhibitors [4]. A-type PAC oligomers, found uniquely in high amounts in cranberries, are characterized by the presence of A-type interflavan double-linkage (see arrow in Fig 1) that together with the specific degree-of-polymerization (DP 4–13) appear to be essential for inhibition of insoluble glucans synthesis by surface-GtfB and -GtfC due to enhanced protein-polyphenol interaction [19,21,34]. B-type PACs (linked by a single interflavan bond) present in other fruits [35] and smaller cranberry PAC dimers/trimers are either devoid or exhibit less inhibitory effects than A-type PAC oligomers [19,20]. Conversely, the inhibitory effects of myricetin may be associated with the presence of a single double-bound between C2 and C3 (Fig 1) and/or a ketone oxygen at the C4 position in contrast to other cranberry polyphenols, such as anthocyanins, which have a fully unsaturated C ring and lack the C4 ketone oxygen, are unable to affect Gtf activity [17,36].

The anti-Gtf activity of the selected cranberry-derived flavonoids combined with inhibitory effects of A-type PAC on *S*. *mutans* adhesion to glucan-coated surfaces [20] explains at least in part the efficacy of CranFlav in hindering the assembly of an insoluble EPS-matrix and microcolony development. The establishment of EPS-enmeshed microcolonies is a critical sucrose-dependent mechanism that favors *S*. *mutans* colonization and further accumulation within biofilms as this bacterium not only produces Gtfs but also expresses multiple EPS (glucan)-binding proteins [37,38]. Such effects not only reduce *S*. *mutans* dominance but can also help to disrupt the mechanical stability of the biofilms as observed in this study. EPS provides a continuum of bacterial binding sites that increases *S*. *mutans* adhesion strength to apatitic surfaces [29]. Furthermore, the presence of an EPS-rich matrix and microcolonies are critical for the biofilm physical integrity and attachment strength at the pellicle interface [10,14,39,40].

The presence of firmly-anchored microcolonies can facilitate the onset of dental caries as highly acidic pH values (4–4.5) are detected at the microcolony/apatite interface due to metabolic activity of the densely packed bacteria enmeshed within a diffusion-limiting EPS matrix [7,10]. Thus, biofilm architecture devoid of microcolonies and reduced EPS content could explain the elevated pH at the biofilm/sHA interface in CranFlav-treated biofilms. The critical pH for demineralization of enamel appears to be as low as 5.1 in the plaque fluid as recently reviewed [30]. Therefore, it is possible that CranFlav could also interfere with the initiation and progression of carious lesions.

Collectively, our data reveal the potential of cranberry flavonoids to prevent dental caries by disrupting major cariogenic properties of the biofilm without displaying bactericidal activity, which support the concept of controlling virulent biofilms without killing [25]. CranFlav are retained at the sHA/biofilm interface despite brief, topical-exposure regimen, and caused major changes in the biofilm composition by primarily targeting Gtf activity [19,21] and *S*. *mutans* adhesion to Gtf-derived glucans [20]. The amount of insoluble EPS was substantially reduced (>80% reduction), while compromising *S*. *mutans* accumulation within mixed-species biofilms. In turn, the 3D biofilm architecture was severely compromised, facilitating its mechanical removal. Furthermore, biofilms treated with cranberry bioactives showed significantly higher pH values at the biofilm-apatite interface. These observations provide important insights on how cranberry-derived flavonoids can modulate cariogenic biofilms, while laying out a framework for further *in vivo* efficacy studies that may lead to new adjunctive antibiofilm-anticaries chemotherapeutic formulations.

## Supporting Information

S1 Fig Retention of cranberry flavonoids in biofilms. The amount of cranberry flavonoids retained within the biofilms was estimated on the determination of total phenolic using Folin-Denis method. Briefly, dry biofilm pellet was dissolved in 50% methanol (1 mg biofilm dry-weight/mL) and subjected to ultrasonic-assisted extraction (ultrasonic bath for 10 min following probe sonication for 30 sec) followed with overnight incubation at room temperature to improve the solubility of bound phenolic compounds. An aliquot of extract (0.2 mL) was mixed with 1.8 mL of MilliQ-water and 0.2 mL of Folin-Denis reagent was subsequently added. The mixture was vortexed vigorously and allowed to stand for 3 min. Then, 0.4 mL of 10% Na_2_CO_3_ and 1.4 mL of MQ-water were added. After 1 h at room temperature, the reaction mixture was analyzed spectrophotometrically using the absorbance at 725 nm to detect the reduction of phosphotungstomolydbdic acid by phenolic compounds (blue color). The amount of total phenolics is expressed as tannic acid equivalents (μg tannic acid per mg of biofilm).
(TIF)

S2 Fig Effects of selected cranberry flavonoids on surface-adsorbed GtfB activity and *S*. *mutans* accumulation within biofilms. (A) The influence of cranberry flavonoids alone or in combination on the activity of Gtf B adsorbed onto a salivary-coated hydroxyapatite (sHA) surface was determined. Briefly, GtfB adsorbed to sHA beads were mixed with each of the test agents or vehicle control (20% EtOH and 0.8% DMSO), and then washed to remove excess or unbound material. Then, the treated surface-GtfB was incubated with [^14^C]glucose labeled-sucrose at 37°C for 4 h, and the amount of Gtf activity was measured by scintillation counting. The concentrations of cranberry flavonoids were 300 μM (DP4), 100 μM (DP9), and 2 mM (myricetin). (B) The proportion of *S*. *mutans* in the mixed-species biofilms was calculated based on total and *S*. *mutans* viable cell (colony forming units; CFU) counting within biofilm. DP, a degree-of-polymerization; Myr, myricetin.
(TIF)

S3 Fig Effect of CranFlav on the bacterial cell viability in planktonic cells and in mixed-species biofilms. (A) Viability of planktonic cells of *S*. *mutans* (10^5^ CFU/ml) after incubation with CranFlav or vehicle control for 5 min exposure. (B) The total viable cell population in the biofilms (91 h) treated with CranFlav or vehicle control.
(TIF)

S4 Fig Representative 3D-randered images of mixed-species biofilms at 67 h. Biofilms were treated with the vehicle control (A) or with CranFlav (B). The bacterial cells are in green and EPS are in red. Scale bar = 100 μm.
(TIF)
