# Supplementary figures and images for: Cranberry Flavonoids Modulate Cariogenic Properties of Mixed-Species Biofilm through Exopolysaccharides-Matrix Disruption
Source: PLoS One. 2015 Dec 29;10(12):e0145844. doi: 10.1371/journal.pone.0145844 (PMC4699891; doi:10.1371/journal.pone.0145844)

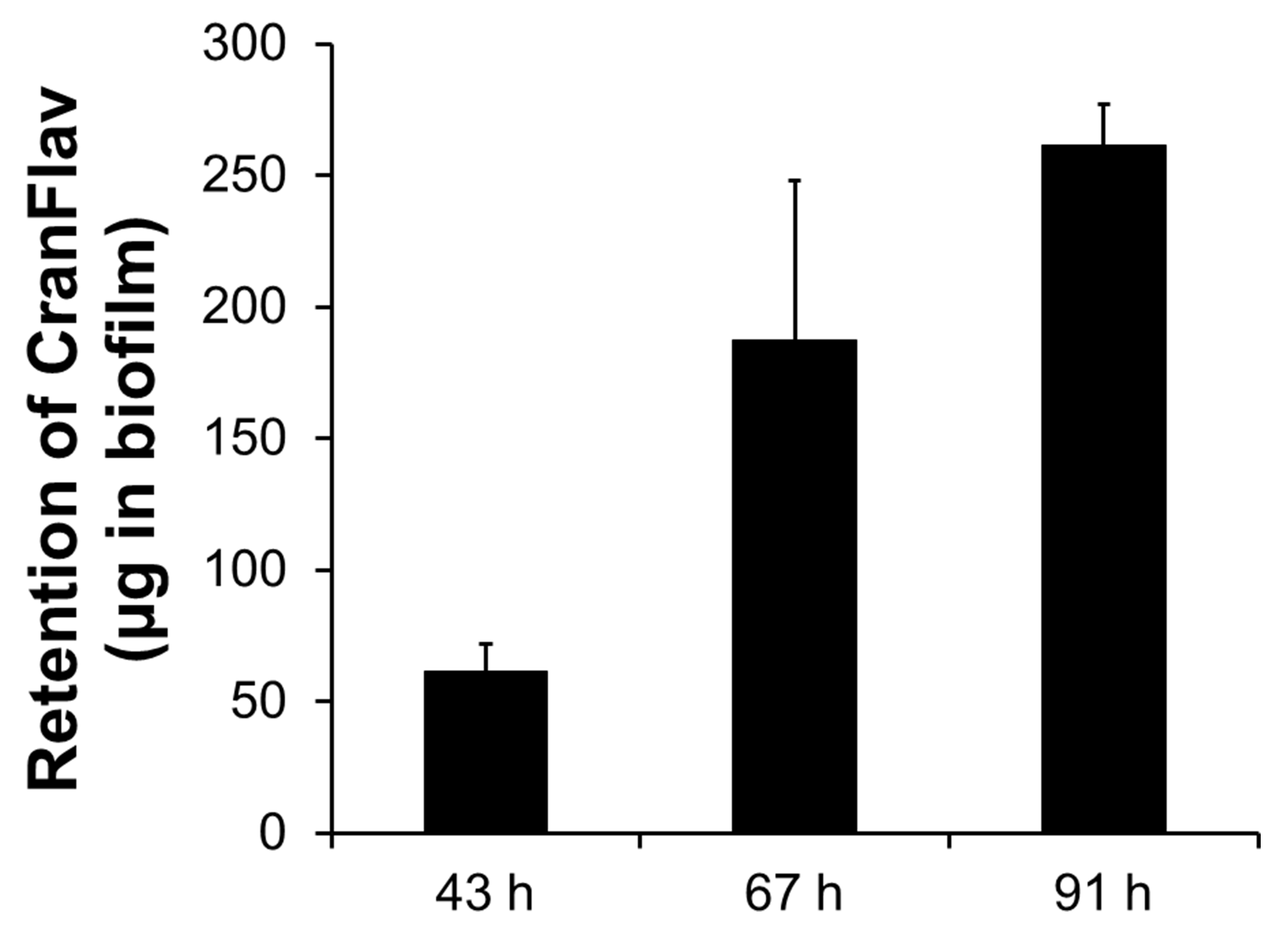

Supplement: S1 Fig — The amount of cranberry flavonoids retained within the biofilms was estimated on the determination of total phenolic using Folin-Denis method. Briefly, dry biofilm pellet was dissolved in 50% methanol (1 mg biofilm dry-weight/mL) and subjected to ultrasonic-assisted extraction (ultrasonic bath for 10 min following probe sonication for 30 sec) followed with overnight incubation at room temperature to improve the solubility of bound phenolic compounds. An aliquot of extract (0.2 mL) was mixed with 1.8 mL of MilliQ-water and 0.2 mL of Folin-Denis reagent was subsequently added. The mixture was vortexed vigorously and allowed to stand for 3 min. Then, 0.4 mL of 10% Na2CO3 and 1.4 mL of MQ-water were added. After 1 h at room temperature, the reaction mixture was analyzed spectrophotometrically using the absorbance at 725 nm to detect the reduction of phosphotungstomolydbdic acid by phenolic compounds (blue color). The amount of total phenolics is expressed as tannic acid equivalents (μg tannic acid per mg of biofilm). (TIF) [file pone.0145844.s001.tif]

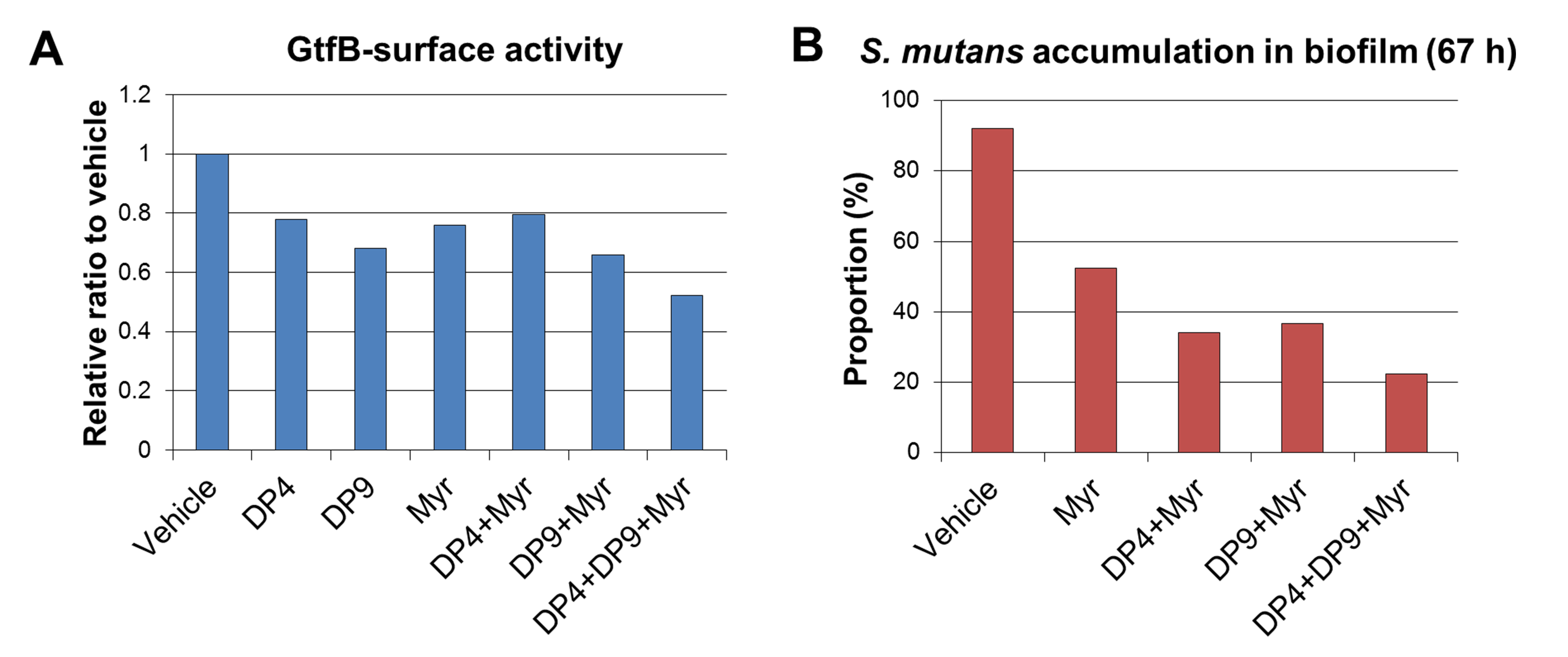

Supplement: S2 Fig — (A) The influence of cranberry flavonoids alone or in combination on the activity of Gtf B adsorbed onto a salivary-coated hydroxyapatite (sHA) surface was determined. Briefly, GtfB adsorbed to sHA beads were mixed with each of the test agents or vehicle control (20% EtOH and 0.8% DMSO), and then washed to remove excess or unbound material. Then, the treated surface-GtfB was incubated with [14C]glucose labeled-sucrose at 37°C for 4 h, and the amount of Gtf activity was measured by scintillation counting. The concentrations of cranberry flavonoids were 300 μM (DP4), 100 μM (DP9), and 2 mM (myricetin). (B) The proportion of S. mutans in the mixed-species biofilms was calculated based on total and S. mutans viable cell (colony forming units; CFU) counting within biofilm. DP, a degree-of-polymerization; Myr, myricetin. (TIF) [file pone.0145844.s002.tif]

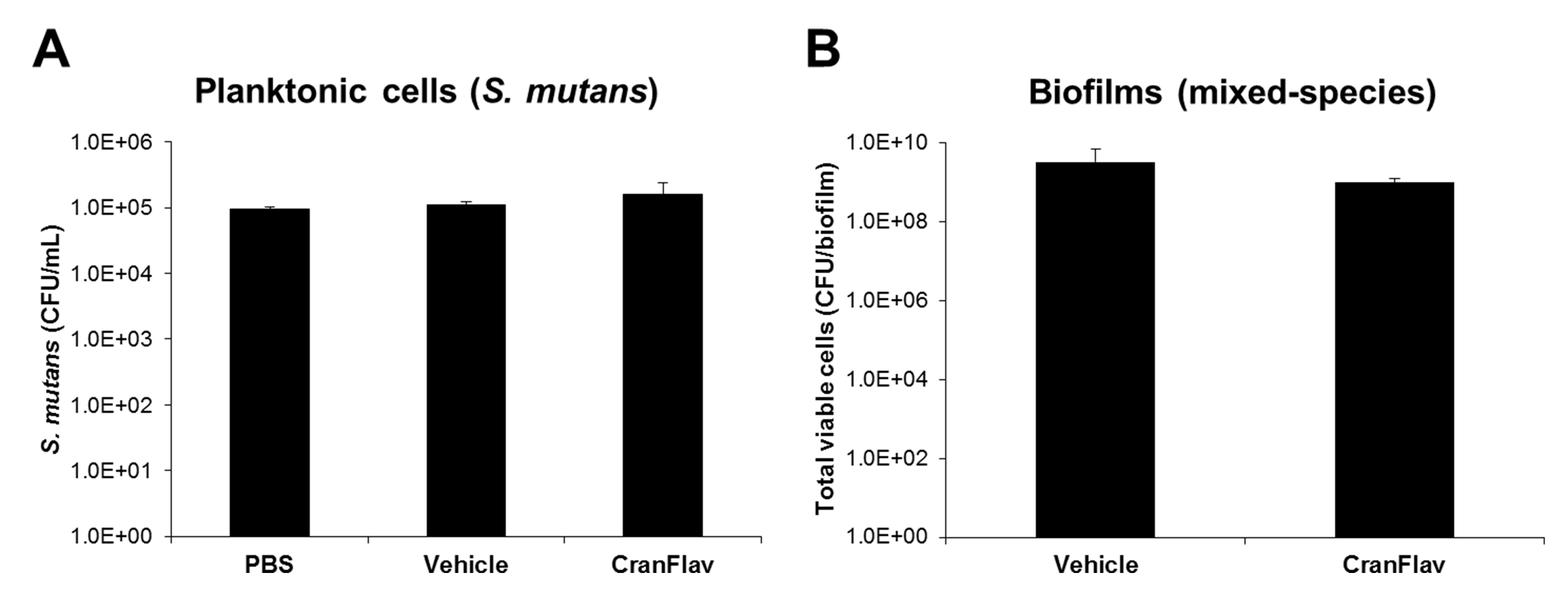

Supplement: S3 Fig — (A) Viability of planktonic cells of S. mutans (105 CFU/ml) after incubation with CranFlav or vehicle control for 5 min exposure. (B) The total viable cell population in the biofilms (91 h) treated with CranFlav or vehicle control. (TIF) [file pone.0145844.s003.tif]

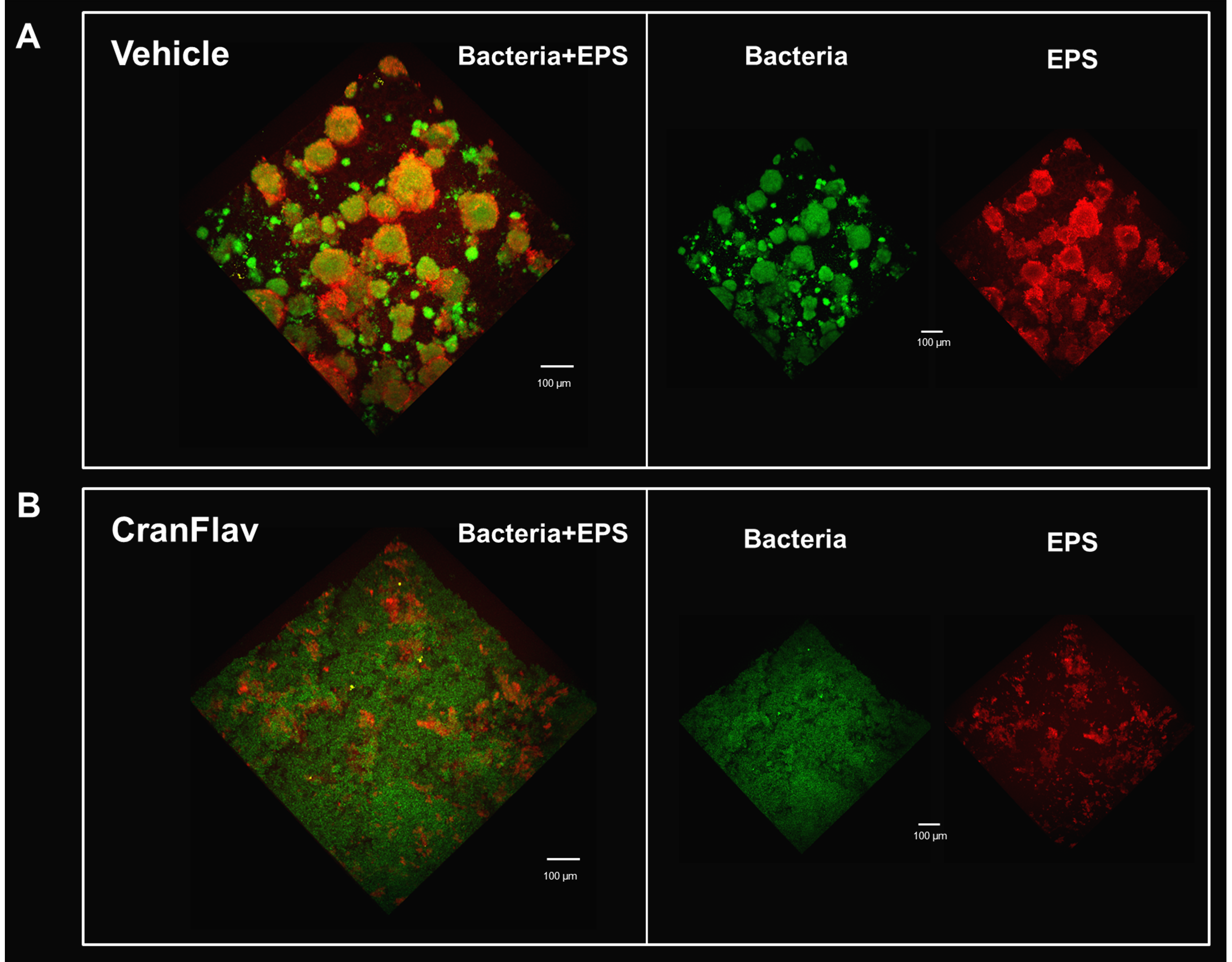

Supplement: S4 Fig — Biofilms were treated with the vehicle control (A) or with CranFlav (B). The bacterial cells are in green and EPS are in red. Scale bar = 100 μm. (TIF) [file pone.0145844.s004.tif]
